# Adaptative evolution of the *Vkorc1* gene in *Mus musculus domesticus* is influenced by the selective pressure of anticoagulant rodenticides

**DOI:** 10.1002/ece3.2829

**Published:** 2017-03-21

**Authors:** Joffrey Goulois, Véronique Lambert, Lionel Legros, Etienne Benoit, Virginie Lattard

**Affiliations:** ^1^ USC 1233 RS2GPVetAgro Sup, INRA, Univ LyonF‐69280MARCY L’ETOILEFrance; ^2^ Liphatech, BonnelPont du CasseFrance

**Keywords:** anticoagulant rodenticides, *Mus musculus domesticus*, mutations, selection pressure, vitamin K epoxide reductase 1

## Abstract

Anticoagulant rodenticides are commonly used to control rodent pests worldwide. They specifically inhibit the vitamin K epoxide reductase (VKORC1), which is an enzyme encoded by the *Vkorc1* gene, involved in the recycling of vitamin K. Therefore, they prevent blood clotting. Numerous mutations of *Vkorc1* gene were reported in rodents, and some are involved in the resistant to rodenticides phenotype. Two hundred and sixty‐six mice tails were received from 65 different locations in France. Coding sequences of *Vkorc1* gene were sequenced in order to detect mutations. Consequences of the observed mutations were evaluated by the use of recombinant VKORC1. More than 70% of mice presented *Vkorc1* mutations. Among these mice, 80% were homozygous. Contrary to brown rats for which only one predominant *Vkorc1* genotype was found in France, nine missense single mutations and four double mutations were observed in house mice. The single mutations lead to resistance to first‐generation antivitamin K (AVKs) only and are certainly associated with the use of these first‐generation molecules by nonprofessionals for the control of mice populations. The double mutations, probably obtained by genetic recombination, lead to in vitro resistance to all AVKs. They must be regarded as an adaptive evolution to the current use of second‐generation AVKs. The intensive use of first‐generation anticoagulants probably allowed the selection of a high diversity of mutations, which makes possible the genetic recombination and consequently provokes the emergence of the more resistant mutated *Vkorc1* described to date.

## 1 Introduction

**Figure 1:**
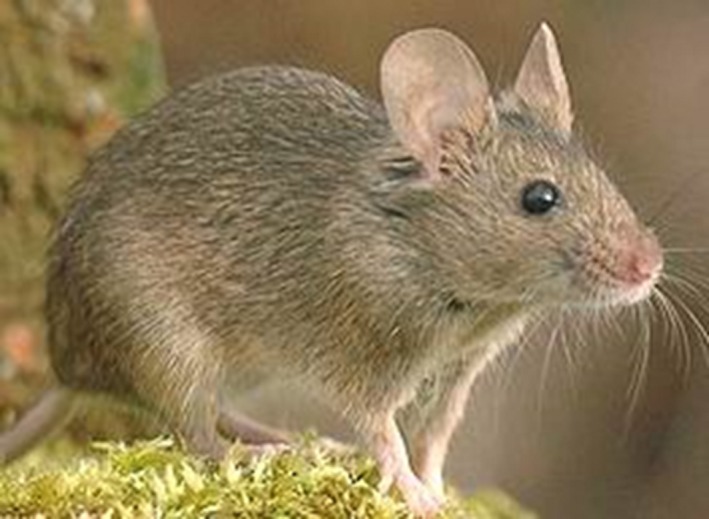
*Mus musculus* domesticus

House mice (Figure 1) are the most widespread mammals on the earth and are present in every continents and all environments (rural, urban, and insular; Angel, Wanless, & Cooper, 2009). *Mus musculus domesticus* belongs to the list of the UICN as one of the 100 most invasive species in the world. To manage rodent populations, chemical controls have been organized since 1950 using antivitamin K (AVK) anticoagulant rodenticides. The intensive use of such molecules for pest control has selected many resistant strains of rodents. Resistance was first detected in brown rats in 1958 (Boyle, 1960) and in house mice in the early 1960s (Dodsworth, 1961) in the UK. Since this initial observation, resistance has been reported worldwide, in many European countries, in the US (Jackson & Kaukeinen, 1972), in Canada (Siddiq & Blaine, 1982), in Japan (Tanaka et al., 2012), and in Australia (Saunders, 1978). The emergence of such resistance to anticoagulants belonging to the first generation (i.e., warfarin, diphacinone, coumatetralyl, chlorophacinone) led to the development of new AVK belonging to the second‐generation molecules (i.e., bromadiolone, difenacoum, flocoumafen, brodifacoum, and difethialone) in the 1970s and 1980s. Nevertheless, the use of such molecules, excessively persistent, exacerbated the risk of primary and secondary poisoning of nontarget species (Caloni, Cortinovis, Rivolta, & Davanzo, 2016; Hughes, Sharp, Taylor, Melton, & Hartley, 2013; Jacquot et al., 2013). Therefore, such molecules should be carefully used.

The resistance to AVKs was proposed to be supported by two major mechanisms in rodents 1/a metabolic resistance due to an accelerated detoxification system involving cytochrome P‐450 (Ishizuka et al., 2007; Sugano et al., 2001) and 2/a target resistance due to the inefficiency of AVKs to specifically inhibit the vitamin K epoxide reductase (VKOR) activity. This VKOR activity is involved in the recycling of vitamin K by allowing the reduction in vitamin K epoxide in vitamin K quinone. Vitamin K is necessary for the activation of clotting factors II, VII, IX, and X. Inhibition of VKORC1 enzyme by AVK molecules results in the absence of gamma‐carboxylated clotting factors II, VII, IX, and X and thus compromises the coagulation process.

While the VKOR activity was described in the 1970s, the VKORC1 enzyme catalyzing this activity was identified in 2004 only by two different teams (Li et al., 2004; Rost et al., 2004) Rost et al., 2004). This enzyme of 163 amino acids is coded by the *vkorc1* gene. This gene is located on the chromosome 7 in mice and on the chromosome 1 in rats. Single nucleotide polymorphisms of this gene were immediately proposed to be responsible for resistance to AVK (Grandemange et al., 2009; Hodroge, Longin‐Sauvageon, Fourel, Benoit, & Lattard, 2011; Pelz et al., 2005; Rost et al., 2004) and appeared to support the resistance process in western Europe, even if cohabitation of target resistance and metabolic resistance had been demonstrated in Denmark (Markussen, Heiberg, Fredholm, & Kristensen, 2007, 2008).

In this paper, we report the different mutations of *M. musculus domesticus Vkorc1* gene observed in different parts of France, six of these mutations being described for the first time. By the use of recombinant VKORC1, we thus analyzed the catalytic consequences of all the different mutations described to date in house mice in order to evaluate the resistant phenotype associated with these mutations. This characterization allowed us to better understand the origin of the different evolution of the *Vkorc1* gene between rats and mice. The diversity of *Vkorc1* mutations observed exclusively in *M. musculus domesticus* led to the emergence of double mutants described for the first time in this study. These double mutations of the *Vkorc1* gene are associated with severe resistant to all AVK.

## 2 Materials and Methods

### 2.1 Mice tissue sampling

*Mus musculus domesticus* samples were collected from the national network of pest control operators (PCOs) in 27 of 95 departments (French administrative areas) covering all the country. The tails of dead mice were cut, and the samples were sent to the laboratory by mail in individual tubes in 70% alcohol. They were frozen at −20°C until analysis. For each tail, PCO filled a questionnaire indicating the site where the mouse was collected. Sampling was performed by trapping or by collecting mice found dead after chemical control.

### 2.2 *Vkorc1* sequencing

Genomic DNA was extracted from tail sample using the Macherey‐Nagel Nucleo‐spin Tissue extraction kit (Hoerdt, France). Genomic DNA was amplified using specific primers of *Vkorc1* gene. In order to sequence the totality of the *Vkorc1* gene, two sets of primers were used. The sequences of the first primer set sVKOR‐S1 and sVKOR‐AS1 were (5′‐GATTCTTCCCTCCTGTCC‐3′) and (5′‐AGACCCTGTCTCAAAACCTA‐3′), respectively; they were used to amplify mouse *Vkorc1* gene from nucleotide −36 to nucleotide +1,727. The sequences of the second primer set sVKORC1‐S2 and sVKORC1‐AS2 were (5′‐GAAAGCAGAACACTTAGCAGG‐3′) and (5′‐AACCAACAGCAGAATGCAGCC‐3′) respectively; they were used to amplify the mouse *Vkorc1* gene from nucleotide (+)1,252 to nucleotide (+)2,512. Mouse *Vkorc1* amplifications were performed using sVKOR‐S1 and sVKOR‐AS1 or sVKOR‐S2 and sVKOR‐AS2 (10 pmol), Accuprime polymerase (1 unit, Invitrogen, Cergy Pontoise, France) in a 25 μl reaction volume containing 2 μl DNA, 2.5 μl 10× Accuprime buffer and 200 μmol/L of each deoxynucleotide triphosphate. The amplification was performed at 94°C for 3 min followed by 40 cycles at 94°C for 20 s, 64°C for 20 s, 68°C for 50 s, and a final extension step at 68°C for 10 min. The amplified products were sequenced on both strands.

### 2.3 DNA mutation screening assay

The *Vkorc1* exons 1, 2, and 3 from the 250 mouse samples were sequenced (Biofidal, Lyon, France). The sequences were read with BioEdit software, analyzed using clc sequence viewer 7 software and compared to published *M. musculus d*omesticus sequences published in GenBank in order to detect the homozygous mutations. The heterozygous mutations were confirmed by attentive examination of the sequencing electropherograms.

### 2.4 Heterologous expression of wild‐type and mutated *M. musculus domesticus* VKORC1

The coding sequence corresponding to the *M. musculus domesticus* VKORC1 fused with a c‐myc tag via a flexible (GGS)_3_ in its 3′‐extremity was optimized for heterologous expression in yeast and synthetized by GenScript (Piscataway, NJ, USA). The synthetized nucleotide sequence included EcoRI and XbaI restriction sites at its 5′‐ and 3′‐extremities, respectively. This nucleotide sequence was subcloned into pPICZ‐B (Invitrogen) and sequenced on both strands.

Construction of mVKORC1 mutant was carried out using pPICZ‐mVKORC1 as template with the Quick change site‐directed mutagenesis kit (Stratagene) according to the manufacturer's recommendations. Each mutant was checked by sequencing, and thus expressed in *Pichia pastoris* as described by Hodroge et al. (2011, 2012).

### 2.5 Subcellular fractionation of yeast cells

Microsomes were prepared from yeast cells by differential centrifugation. Briefly, yeast cells were resuspended in 50 mmol/L Phosphate Buffer (pH 7.4) containing 1.15% (w/v) of KCl. They were broken with Zircon beads using Dispermat^®^ LC30 (VMA‐GETZMANN, Germany; 30 min–3,500 rpm) continuously at 4°C and further submitted to differential centrifugation a continuously at 4°C. The 100,000 *g* pellet corresponding to the membrane fraction was suspended by Potter homogenization in HEPES–glycerol buffer (50 mmol/L Hepes, 20% glycerol, pH 7.4). Protein concentrations were evaluated by the method of Bradford (1976) using bovine serum albumin as a standard. Microsomal fractions were frozen at −80°C and used for kinetic analysis.

### 2.6 Vitamin K epoxide reductase activity assays and kinetics

Microsomal VKOR activity was assayed as described previously (Bodin et al., 2005; Haniza et al., 2015). Briefly, standard reactions were performed in 200 mmol/L Hepes buffer (pH 7.4) containing 150 mmol/L KCl, 1 mmol/L dithiothreitol, and 1 g/L of total proteins. The reaction was started by the addition of vit K_1_>O solution in 1% Triton X‐100 and incubated at 37°C for 30 min. In these conditions, the reaction was linear according to the time of incubation and the quantity of incubated proteins. After incubation at 37°C for 30 min, the reaction was stopped by adding of 2 ml of isopropanol. After centrifugation at 3,000 *g* for 10 min in order to precipitate proteins, 2 ml of hexane was added. After centrifugation at 3,000 *g* for 10 min, the hexane layer was removed and dried under nitrogen. The dry residue was immediately dissolved in 0.2 ml of methanol, and reaction product was analyzed by liquid chromatography–mass spectrometry.

The LC‐APCI/MS/MS used was a 6120 Quadrupole LC/MS with an atmospheric pressure chemical ionization (APCI) interface and a LCMS Chemstation software from Agilent Technologies (Palo Alto, CA, USA). Chromatographic separation was performed using a XTerra MS C18 column (2.1 mm × 50 mm, 2.5 μm; Waters, Milford, MA, USA) with a mobile phase of methanol, 0.1% acetic acid (96:4) in isocratic condition. The column temperature was 48°C. The flow rate in the LC column was 0.4 ml/min. The injection volume was 20 μl. The temperature of the auto sampler tray was set to 5°C, and the samples were protected from the daylight. Detection was by MS with APCI source in positive mode. Nebulizer pressure was set to 60 psi, dry gas temperature to 350°C, dry gas flow to 5 L/min, and vaporizer temperature to 400°C. Capillary voltage was set to 4,000 V, corona needle to 10 μA. Identification criteria for vit K_1_ are the retention time (tr = 234 s) and the selected ion 451.4. Identification criteria for vit K_1_>O are the retention time (tr = 174 s) and the selected ion 467.0. Linearity and accuracy were tested from 25 to 2,000 ng/ml (*n* = 20). The response was linear throughout the concentration range tested with a coefficient of correlation (*r* ^2^) above .99. Accuracy was between 80% and 120% of the theoretical concentrations.

**Figure 2:**
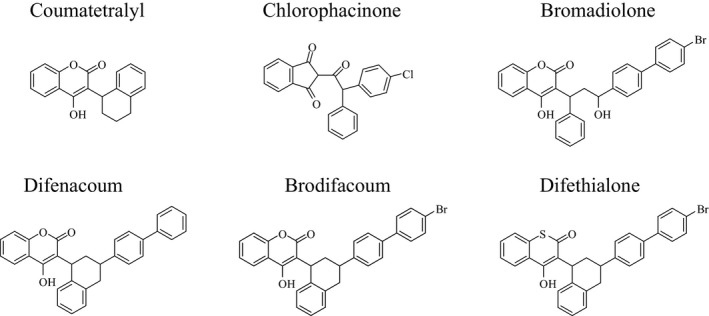
Chemical structures of the AVK compounds used for the inhibition of the wild‐type and mutated VKORC1 expressed in *Pichia pastoris*

*K*_m_, *V*_max,_ and *K*_i_ values were obtained from at least three separate experiments performed on two different batches of protein. The estimation of *K*_m_ and *V*_max_ values was achieved by the incubation of at least nine different concentrations of vit K>O (from 0.003 to 0.2 mmol/L) to the standard reaction. Incubations were performed in duplicate. Data were fitted by nonlinear regression to the Michaelis–Menten model using the R‐fit program. In order to evaluate the inhibiting effect of AVKs on VKOR activity, *K*_i_ were determined after addition of various concentrations of anticoagulant (Figure 2) to the standard reaction in the presence of increasing amounts of vit K>O (from 0.003 to 0.2 mmol/L) using anticoagulant concentrations from about 0.05 to 20 × *K*_i_. Data were fitted by nonlinear regression to the noncompetitive inhibition model *v* = (*V*_max_/(1 + (I/*K*_i_))) × (*S*/(*K*_m_ + *S*)) using the R‐fit program.

## 3 Results

### 3.1 Genotyping results

**Figure 3:**
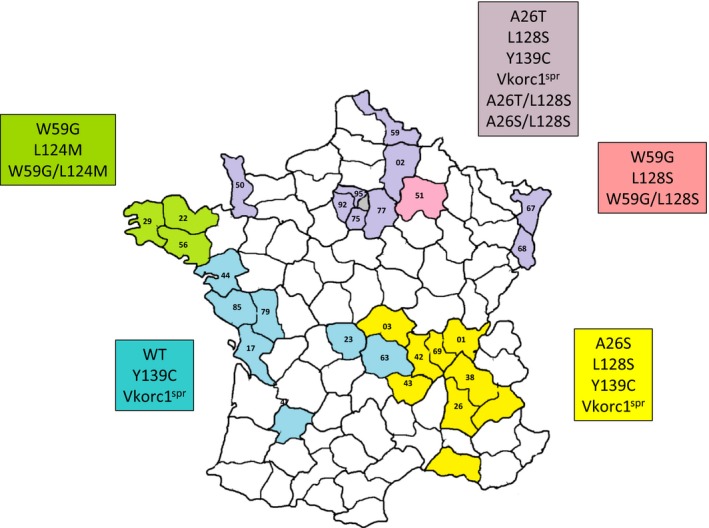
Map of France showing the distribution of *Vkorc1* mutations. Colored areas are areas from where tails of mice were received. Numbers correspond to the French administrative department. French departments are grouped into four geographical areas correlated with Table 2. Brittany is colored in green, North in purple, Marne in pink, South East in yellow, and South West in blue. In the corresponding colored frame are marked mutations observed in this area

A total of 266 tail samples were sent by PCO from 27 administrative departments, indicated by their respective administrative number in Figure 3. Sampling was performed in 65 different locations. Species identification for each sample was based on mitochondrial cytochrome b sequence. For all the samples, amplified sequences presented more than 98% of homology with published sequences of *M. musculus domesticus cytochrome b*.

To identify mutations in the *Vkorc1* gene of the 266 mice, the coding sequences and splice junctions of *Vkorc1* gene were amplified and sequenced. Obtained sequences were subsequently compared to published *Vkorc1* sequences for *M. musculus domesticus* (GenBank accession number GQ905715) and for *Mus spretus* (GenBank accession number GQ905711). Only 73 samples presented no missense mutation in the coding sequences of *Vkorc1* gene (i.e., 27.4% of the mice tails sent by PCO). One hundred and ninety‐three samples presented at least one missense mutation in the coding sequences of *Vkorc1* gene (i.e., 72.6% of the mice tails sent by PCO). From the 193 French mice carriers for *Vkorc1* missense mutations, 80% were homozygous and only 20% were heterozygous.

Ten different mutations were found in the *Vkorc1* gene in the French mice. The descriptions of the mutations found in the *Vkorc1* gene are summarized in Table 1. In exon 1, five mutations were detected. These mutations were located at nucleotide 34 (g.34C>T), 76 (g.76G>T or g.76G>A), 111 (g.111A>G), and 142 (g.142G>C). Except for the g.111A>G mutation, these mutations were missense mutations leading to mutations R12W, A26S, A26T, and A48T, respectively. Two mutations were detected in exon 2; they are located at nucleotide 969 (g.969T>G) and nucleotide 976 (g.976G>T) and led to mutations W59G and R61L, respectively. The W59G was detected only at the homozygous state. Three mutations were detected in exon 3, at nucleotide 2177 (g.2177C>A), 2190 (g.2190T>C), and 2223 (g.2223A>G) and conducted to mutations L124M, L128S, and Y139C, respectively.

**Table 1:** Detail of SNPs and mutations of *Vkorc1* found in French mice

Mutation	Nucleotide position	Codon WT	Codon mut	AA WT	AA mut	Exon
R12W	34	CGG	TGG	Arg	Trp	1
A26S	76	GCA	TCA	Ala	Ser	1
A26T	76	GCA	ACA	Ala	Thr	1
E37E	111	GAA	GAG	Glu	Glu	1
A48T	142	GCC	ACC	Ala	Thr	1
W59G	969	TGG	GGG	Trp	Gly	2
R61L	976	CGG	CTG	Arg	Leu	2
L124M	2,177	CTG	ATG	Leu	Met	3
L128S	2,190	TTA	TCA	Leu	Ser	3
Y139C	2,223	TAT	TGT	Tyr	Cys	3
A26S/L128S	76	GCA	TCA	Ala	Ser	1
	2,190	TTA	TCA	Leu	Ser	3
A26T/L128S	76	GCA	ACA	Ala	Thr	1
	2,190	TTA	TCA	Leu	Ser	3
W59G/L124M	969	TGG	GGG	Trp	Gly	2
	2,177	CTG	ATG	Leu	Met	3
W59G/L128S	969	TGG	GGG	Trp	Gly	2
	969	TGG	GGG	Trp	Gly	2

Twelve *Vkorc1* genotype were observed in the French mice. Six *Vkorc1* genotypes detected in the French mice led to a single mutation in the corresponding VKORC1 protein (i.e., A26T, A26S, W59G, L124M, L128S, or Y139C). The locations of these genotypes are presented in Figure 3. The observed allelic frequencies of A26T, A26S, W59G, L124M, L128S, and Y139C in our sampling, were, respectively, 1.3%, 0.4%, 1.1%, 3.4%, 14.9%, and 12.3%. The allelic frequencies of these mutations were different between geographical areas. The results are summarized in Table 2 with the number of samples and the number of mutated samples. The departments were gathered according to five geographical areas (i.e., Brittany, North, Marne, South West and South East) presented in Figure 3. Mice carriers for two of these mutations, the A26T and Y139C mutations (one mouse), but also the L128S and Y139C mutations (four mice) at the heterozygous state, were detected. Cloning and sequencing of the *Vkorc1* gene from these mice revealed that these mutations were present on different alleles.

**Table 2:** Detailed locations and frequencies of SNPs and mutations of *Vkorc1* found in French mice

Area	No. of sampling sites	No. of mice	No. of mutated mice	% of mutation/area	Mutation	HeteroZ	HomoZ	Allelic frequency
Brittany	13	31	19	61	W59G	0	2	6.5
					L124M	4	7	29
					W59G + L124M	0	6	19.4
North	17	82	74	90	A26T	1	3	4.3
					L128S	7	28	38.4
					A26T + L128S	3	14	18.9
					Y139C	7	4	9.1
					*Vkorc1* ^spr^	0	7	8.5
					A26S + R61L	0	1	1.2
					A26S + L128S	0	2	2.4
Marne	1	6	5	83	W59G	0	2	33
					L128S	2	0	17
					W59G + L128S	0	1	17
South West	13	29	6	20	Y139C	1	4	15
					*Vkorc1* ^spr^	1	0	1.7
South East	21	118	89	75	A26S	0	1	0.9
					L128S	7	4	6.4
					Y139C	8	18	18.6
					*Vkorc1* ^spr^	3	50	43.6

The other genotypes led to VKORC1 with two or more mutations at the homozygous state. The combination of R12W, A26S, and A48T in exon 1 associated with the R61L mutation in exon 2 was previously described as the *Vkorc1* ^spr^ genotype (Song et al., 2011). However, other mutations were found to be present on the same allele of *Vkorc1*. The g.969T>G mutation was found to be associated with the g.2177C>A mutation or the g.2190T>C mutation at the homozygous state leading to proteins with two combined mutations, the W59G and L124M or the W59G and L128S. The allelic frequencies of these two genotypes in our sampling were, respectively, 2.3% and 0.4% in France. The association of the g.969T>G mutation with the g.2177C>A mutation was only found in Brittany (Table 2 and Figure 3), and the association of the g.969T>G mutation with the g.2190T>C mutation was only found in Marne (Table 2 and Figure 3). The g.76G>T mutation was found to be associated with the g.976G>T mutation or the g.2190T>C mutation at the homozygous state leading to proteins with two combined mutations, the A26S and R61L or the A26S and L128S. The observed allelic frequencies of these two genotypes in our sampling were, respectively, 0.4% and 0.8% in France. Both genotypes were found in the North of France (Table 2 and Figure 3). The g.76G>A mutation was found to be associated with the g.2190T>C mutation at the homozygous state leading to a protein with two combined mutations, the A26T and L128S. The allelic frequency of this genotype in our sampling was 5.3%, and this genotype was detected only in the North of France.

### 3.2 Functional consequences of VKORC1 mutations

To assess the consequences of VKORC1 mutations on the functional properties of VKORC1, wild‐type VKORC1 and its mutants were overexpressed as c‐myc‐fused proteins in *P. pastoris*. All the French single and double mutants, but also mutants detected in other countries in previous studies, were characterized in this study. All proteins were efficiently expressed in *P. pastoris* with the same expected molecular mass of approximately 20 kDa.

The ability of each membrane protein to catalyze the reduction of K>O to K was determined. Five single or double mutants (i.e., W59G, W59L, W59S, W59G/L124L, and W59G/L128S) presented less than 2% of the VKOR activity determined for wild‐type VKORC1 preventing additional studies (Table 3). The other mutants were all able to reduce the vitamin K epoxide with *K*_m_ similar to wild‐type VKORC1 (Table 3).

**Table 3:** Apparent kinetic parameters toward vit K_1_>O obtained for yeast microsomes expressing wild‐type or mutated vitamin K epoxide reductase 1 (VKORC1)

Mutations	*K*_m_ (μM)
WT	28 ± 3
R12W	49 ± 29
A26S	21 ± 8
A26T	7 ± 0.5^a^
E37G	66 ± 18
A48T	38 ± 20
R58G	45 ± 19
W59G	VKOR activity < 2% of WT
W59L	VKOR activity < 2% of WT
W59S	VKOR activity < 2% of WT
R61L	42 ± 22
L124M	11 ± 8^a^
L124Q	20 ± 7
L128S	18 ± 5
Y139C	35 ± 1
A26T/L128S	52 ± 2.5^a^
A26S/L128S	31 ± 4
W59G/L124M	VKOR activity < 2% of WT
W59G/L128S	VKOR activity < 2% of WT
*Vkorc1* ^spr^	25 ± 5

To determine the VKOR activity, standard reactions were performed in 200 mmol/L Hepes buffer (pH 7.4) containing 150 mmol/L KCl and 0.25–2 g/L of microsomal proteins expressing membrane wild‐type or mutant VKORC1. Each data point represents the mean ± *SD* of three individual determinations. Comparison between two groups was made using Mann–Whitney test.

a *p* < .05 compared to wild‐type VKORC1.

**Figure 4:**
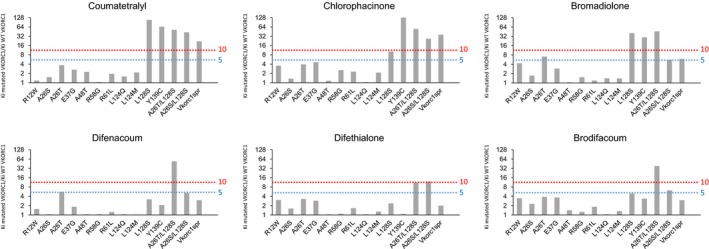
Inhibition effect of various generation antivitamin K on mutated vitamin K epoxide reductase 1 (VKORC1) expressed in yeast microsomes, comparatively to the *Mus musculus* VKORC1^WT^

In order to compare the susceptibilities to AVKs of wild‐type VKORC1 and its mutants, their respective inhibition constants *K*_i_ toward various AVKs of the first generation (i.e., coumatetralyl, chlorophacinone) or the second generation (i.e., bromadiolone, difenacoum, difethialone, or brodifacoum) were determined. All AVKs were able to inhibit the VKOR activity in a noncompetitive manner for all the mutants. Nevertheless, the concentrations of AVKs necessary to inhibit the VKOR activity were different between mutants and between inhibitors. Results are presented in Figure 4 as a ratio between the *K*_i_ obtained for the mutated protein and the *K*_i_ obtained for the wild‐type protein, this ratio representing the resistance factor of the mutated protein.

## 4 Discussion

All the 15 missense mutations in the mice *VKorc1* observed all over Europe have been characterized using the same method which was also previously used to characterize the catalytic consequences of the human (Hodroge et al., 2012) and the rat *Vkorc1* mutations (Hodroge et al., 2011). The corresponding recombinant proteins were expressed in *P. pastoris*, and their catalytic properties were determined. Resistance factors corresponding to the ratio between *K*_i_ obtained for the mutated VKORC1 and for the wild‐type VKORC1 were determined for each VKORC1 protein toward each anticoagulant rodenticide. These in vitro resistance factors used for the first time to study the consequences of the *Vkorc1* mutations in brown rats (Hodroge et al., 2011) were shown to be totally coherent with resistance factors obtained using liver microsomes (Lasseur et al., 2005) and with resistance factors obtained by BCR tests after *peros* administration of AVK to strains of rats homozygous for wild‐type *Vkorc1* or *Vkorc1‐Y139F* (Grandemange et al., 2009) or by feeding tests in mice homozygous for wild‐type *Vkorc1* or *Vkorc1* ^spr^ (Goulois et al., 2017). Therefore, in this study, the resistance factors determined in vitro using recombinant VKORC1 expressed in yeast are considered as good markers of the in vivo resistance phenotype of rodents, for whom we possessed only fragments of tail.

Among the *Vkorc1* mutations detected in mice, two single missense mutations and four double mutations have never been described (A26T, L124M, A26T/L128S, A26S/L128S, W59G/L124M, W59G/L128S). Different mutations (i.e., A26T, E37G, R58G, L124M, and L124Q) lead to a moderate resistance (Figure 4) to first‐generation AVK such as chlorophacinone and coumatetralyl with resistance factors of about 5; second‐generation molecules remaining still efficient. Such resistance factors allow certainly mice homozygous for one of these mutations to survive when pest control is performed using first‐generation AVK. On the contrary, the use of second‐generation AVK will eliminate such mice. On the other hand, *Vkorc1* mutations observed in mice such as L128S, Y139C, or the *Vkorc1* ^spr^ genotype lead to a severe resistance to first‐generation AVK, but also confer a limited resistance to some second‐generation AVK.

The genetic variation of the *Vkorc1* gene of mice in terms of number of missense mutations appears to be similar to that described in humans and rats. Indeed, mutations of the *Vkorc1* gene have been particularly analyzed in humans because of the massive therapeutic use of AVK worldwide in human, with almost 22 million of patients receiving daily AVK and because of the wide variability of dosage necessary to achieve stable anticoagulation between patients. This variability being partly due to genetic polymorphisms in the *Vkorc1* gene, numerous studies have been performed to detect new *Vkorc1* mutations in patients resistant to AVK. These studies allowed to detect in humans 27 mutations in the coding sequence of the *Vkorc1* gene (Bodin, Horellou, Flaujac, Loriot, & Samama, 2005; Bodin, Perdu, Diry, Horellou, & Loriot, 2008; D'Andrea et al., 2005; Harrington, Siddiq, Allford, Shearer, & Mumford, 2011; Harrington et al., 2008; Loebstein et al., 2007; Osman, Enström, Arbring, Söderkvist, & Lindahl, 2006; Peoc'h, Pruvot, Gourmel, Dit Sollier, & Drouet, 2009; Rieder et al., 2005; Rishavy, Usubalieva, Hallgren, & Berkner, 2011; Rost et al., 2004; Schmeits et al., 2010; Watzka et al., 2011; Wilms, Touw, Conemans, Veldkamp, & Hermans, 2008). In rats, 15 missense mutations in the *Vkorc1* gene of *Rattus norvegicus* have been described in Europe (Grandemange, Lasseur, Longin‐Sauvageon, Benoit, & Berny, 2010; Haniza et al., 2015; Pelz et al., 2005, 2012; Rost et al., 2004). The number of mutations reported in this gene is thus comparable between rats, mice, and humans. Therefore, the existence of such a diversity of mutations in the *Vkorc1* gene cannot be associated with the management of rodent populations by the intensive use of AVK molecules while on the contrary the frequency of these mutations is clearly linked to this massive AVK use for pest control.

In humans, the allelic frequencies of *Vkorc1* mutations are well below 1% and the resistance levels to AVK conferred by all the mutations are limited (Hodroge et al., 2012) compared to that conferred by some *Vkorc1* mutations in rats (Hodroge et al., 2011). Indeed, the use of warfarin or other AVK in humans cannot be considered as a selection pressure as the average age of patients treated with AVK is around 70 years.

In rats, if 15 *Vkorc1* mutations have been reported in Europe, only three mutations are frequently and mostly observed in Europe. In France and Belgium, the major mutation observed in the *Vkorc1* gene of brown rats is the Y139F mutation (Baert, Stuyck, Breyne, Maes, & Casaer, 2012; Grandemange et al., 2010), the observed allelic frequency of the Y139F mutation was reported in 2010 to be 21% from a sampling of 268 rats trapped from 91 sites well distributed in France (Grandemange et al., 2010), while the other mutations found in France (i.e., Y139C, L128Q, L120Q, E155K, S103Y) accounted for only 6.5% of the *Vkorc1* alleles analyzed. The repetition of this resistance monitoring conducted between 2014 and 2015 by our laboratory suggests an increase in the allelic frequency of the Y139F in France since 2010, with an observed allelic frequency that has now reached over 60% in a population of 180 rats trapped in 51 sites from 18 French administrative departments (data not shown). Moreover, this allelic frequency can locally reach levels close to 80% in rural areas (Berny, Fourel, & Lattard, 2014). In Germany, the Netherlands, and England, the predominance of one mutation in brown rats populations seems also to be the case with the Y139C mutation distributed in foci of resistance at frequencies similar to that observed in France for the Y139F mutation (Haniza et al., 2015; Meerburg, van Gent‐Pelzer, Schoelitsz, & van der Lee, 2014; Pelz, 2007). Nevertheless, as described for France, other *Vkorc1* mutations can be detected in Germany and England, but with allelic frequency very low. Contrary to humans, the frequency of the *Vkorc1* mutations in brown rats is linked to the selection pressure due the intensive use of AVK to control rat populations. Despite increased vitamin K requirement associated with Y139F, Y139C, and L120Q *Vkorc1* mutations, these mutations confer for brown rats such a benefit in the field where intensive use of AVKs is done, that they have become widely predominant leading to the almost disappearance of other *vkorc1* mutations in foci of resistance. Indeed, the Y139F, Y139C, and L120Q mutations lead to drastic resistance to AVK of the first generation such as chlorophacinone and coumatetralyl, but also to some AVK of the second generation such as bromadiolone and to a lesser extent difenacoum (Hodroge et al., 2011). During pest control management with one of these molecules, only the brown rats carrier for one of these mutations, in the homozygous state and also, but to a lesser extent, in the heterozygous state, survive. Nevertheless, brown rats carriers of one of these mutations are still susceptible to AVK such as difethialone, brodifacoum, and flocoumafen and control with one of these molecules is still possible (Hodroge et al., 2011; Lasseur et al., 2005).

In mice, 15 mutations have been detected in Europe in this study and in a previous study performed in 2012 (Pelz et al., 2012). Frequency of mice carriers for *Vkorc1* mutations was found to be extremely elevated in both studies. In the previous study performed in Germany, Switzerland, and Azores, 80% of mice trapped from 30 sites were found to be carrier of one mutation in the *Vkorc1* gene. Only one site was described to be free of mice carrier for *Vkorc1* mutations. In this study, more than 70% of mice trapped from 65 sites were found to be carrier at least of one mutation in the *Vkorc1* gene. Only five sites were found to be free of mice carrier for *Vkorc1* mutations. The method of sampling used in this study could have introduced some biases. Indeed, sampling was performed by PCO who might have preferred to collect mice in hot spots of control problems, which would overestimate the detection of resistances. Nevertheless, this bias could be compensated by the capture method used. The majority of the samples come from mice found dead after chemical control and thus not resistant to AVKs.

In France, nine different *Vkorc1* single missense mutations are observed in house mice that are either spontaneous mutations or mutations due to introgression of the *Vkorc1* gene from *Mus spretus* in the genome of *M. musculus domesticus*. All these mutations can be observed in the homozygous state which demonstrates that very high allelic frequencies can be reached locally for all of them. Contrary to the brown rat populations, while the frequency of mice carrier for *Vkorc1* mutation seems greater, no predominant mutation is observed in the French mice suggesting a different evolution of the *Vkorc1* mutations in mice as compared to brown rats.

In the same territory, Europe, 15 mutations are observed in mice with high frequencies contrary to brown rats for whom only three mutations are observed with high frequency. For a more limited territory, France, nine missense single mutations are observed in mice and one is predominant in the brown rat. This difference could be due to a difference in the selection pressure exerted by AVK. Management of mice is essentially realized by nonprofessionals, and we can assume that first‐generation AVK can be used more extensively by nonprofessionals not informed about resistance problems while management of rats is realized more frequently by better trained and better informed PCO. Moreover, the feeding behavior of mice promotes diversity food sources and thereby induces a limitation of the amount of ingested bait. To that extent, *Vkorc1* mutations conferring even limited in vitro resistance factors to first‐generation AVK have been selected (i.e., A26T, E37G, R58G, L124M and L124Q). Therefore, the preservation of such a diversity of mutations observed in the three exons, (while the three main mutations in the rats concern two codons only and are in the same exon) and the high prevalence of each of these mutations in the homozygous state are responsible for the emergence of double mutants (*i.e*., A26T/L128S, A26S/L128S, W59G/L124M, W59G/L128S) due certainly, to genetic recombination between mutated alleles. Indeed, in the geographical areas where double mutations on the same *Vkorc1* allele were observed, mice carrying the corresponding single mutations in the homozygous state were systematically present. For example in the Brittany region, mice homozygous for the W59G mutation, mice homozygous for the L124M mutation, and mice homozygous for the W59G/L124M double mutations are found and within the same site of capture, the three types of genome were also encountered.

**Figure 5:**
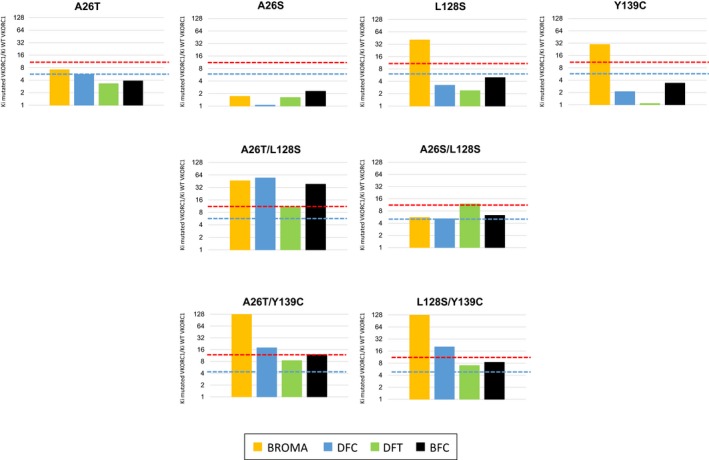
Comparative effect of single and corresponding double *Vkorc1* mutation(s) on the susceptibility to second‐generation generation antivitamin K

The emergence of the double mutations (A26T/L128S, A26S/L128S) confer an evident benefit for mice carrier for these double mutations compared to mice carrier for the corresponding single mutations (Figure 5). While the single A26T, A26S, or L128S mutations lead to moderate resistance to first‐generation AVK, the double mutations lead to resistance to all AVK currently available with resistance factors reaching levels higher than 10 toward difenacoum and brodifacoum and close to 10 toward difethialone for A26T/L128S (Figure 5). Such levels of resistance toward such molecules has never been found for any of the isolated mutations observed in mice, but also in brown rats. The emergence of these double mutations must therefore be regarded as an adaptive response to the use of AVKs. Interestingly, our observation of mice heterozygous for A26T and Y139C or L128S and Y139C on different alleles can be considered as a necessary step prior to the recombination and makes likely the future recombination. Nevertheless, such a recombination has not yet been demonstrated in living mice but their consequences, as shown in Figure 5 would be certainly important.

In this adaptive context, the probable use of the first‐generation AVK for the management of the mice populations and the feeding behavior of this species lead to a high diversity of the mutations in the *Vkorc1* gene and allows, in consequence, the appearance of double mutants very resistant to AVKs. The observation in the field of mice already presenting a clear resistance to the most powerful AVKs (even if this resistance is moderate) is worrying for the control, for the next years, of the mice populations unless if the double mutations in the *Vkorc1* gene are associated with an important biological cost. In brown rats, the intensive use of more efficient second‐generation VKA leads to resistance linked to a very limited number of mutations in the *Vkorc1* gene which correspond to very efficient single missense mutations but which are less efficient than the double mutations observed in mice. The recombination of mutations in rat seems unlikely due to the population structure and the small number of mutations encountered and the predominance of one mutation per geographical area.

## Conflict of Interest

None declared.

## Data Accessibility

Species identification and *Vkorc1* genotype of mice trapped in this study has been deposited on Dryad (doi: 10.5061/dryad.bs888).
